# Prenatal chromosomal microarray analysis in foetuses with isolated absent or hypoplastic nasal bone

**DOI:** 10.1080/07853890.2022.2070271

**Published:** 2022-05-04

**Authors:** Xiaomei Shi, Jian Lu, Ling Li, Ran Wei, Jing Wu

**Affiliations:** Genetic Medical Center, Guangdong Women and Children Hospital, Guangzhou, China

**Keywords:** Absent nasal bone, hypoplastic nasal bone, chromosome microarray analysis, prenatal diagnosis

## Abstract

**Objectives:**

To evaluate the efficiency of chromosomal microarray analysis (CMA) in the prenatal diagnosis of foetuses with isolated absent or hypoplastic nasal bone (NB) in the first and second trimester.

**Methods:**

From January 2015 to April 2021, foetuses with isolated absent or hypoplastic NB who received invasive prenatal diagnosis were enrolled. The results of CMA were analysed

**Results:**

There were 221 foetuses, including 166 cases with isolated absent NB and 55 cases with isolated hypoplastic NB. Twenty-four foetuses (10.9%, 24/221) had an ultrasonic diagnosis in the first trimester and 197 (89.1%, 197/221) had a ultrasonic diagnosis in the second trimester. The overall diagnostic yield of CMA was 9.0% (20/221). Aneuploidies were detected in 13 (5.9%, 13/221) foetuses, including 10 Down syndrome, 2 Klinefelter's syndrome and 1 trisomy 18. Pathogenic copy number variations (CNVs) were detected in seven foetuses (3.2%, 7/221). In addition, variants of unknown significance (VOUS) were detected in four foetuses. The foetuses with isolated absent NB had a higher detection rate of chromosome abnormality than the isolated hypoplastic NB, but the difference was not significant in the statistical analysis (10.2% vs. 5.5%, *χ*^2 ^=0.642, *p* = .423). No significant difference was observed in the detection rate between the first trimester and the second trimester (16.6% vs. 8.1%, *χ*^2^ = 1.002, *p* = .317, Chi-square test).

**Conclusion:**

CMA can increase the diagnostic yield of chromosome abnormality, especially pathogenic CNVs for foetuses with isolated absent or hypoplastic NB. CMA should be recommended when isolated absent or hypoplastic NB is suspected antenatally.7

## Introduction

Absent or hypoplastic foetal nasal bone (NB) found in both first and second trimester has been proven to be one of the strongest markers for Down syndrome [1–3]. Absent or hypoplastic foetal NB is also associated with other common aneuploidies such as trisomy 18, trisomy 13 and Turner syndrome. Rare conditions such as Cri du chat (5p-) syndrome, Wolf-Hirshhorn syndrome (4p-) and Fryns Syndrome have also been reported [4–6].

Chromosomal microarray analysis (CMA), which is capable of simultaneously detecting numerical chromosomal abnormalities and submicroscopic chromosomal imbalances at the whole-genome level, has been applied to identify chromosomal abnormalities in foetuses with structural abnormalities [7–9]. It is well known that if additional anomalies are detected, CMA should be offered to the foetuses with non-isolated absent or hypoplastic foetal NB since the risk of microdeletion/microduplication syndromes is increased [10–13]. However, data on the yield of CMA for isolated absent or hypoplastic foetal NB is controversial. Lostchuck et al. studied 80 cases of isolated hypoplastic foetal NB and showed no cases of pathogenic copy number variations (CNVs) detected [14]. However, recent study with small sample size reported that the rate of pathogenic CNVs was 5.45% (3/55) in the foetuses with isolated absence or hypoplasia NB [15]. As a result, should CMA be offered to isolated absent or hypoplastic foetal NB remain unclear. Therefore, the aim of this study was to evaluate the efficiency of CMA in the prenatal diagnosis of foetuses with isolated absent or hypoplastic NB, to provide further practical evidence for pre-testing consultation.

## Materials and methods

From January 2015 to April 2021, cases with isolated absent or hypoplastic foetal NB detected by ultrasound, whose parents opted to have an invasive prenatal diagnosis by CMA were included in this study. The study was approved by our institutional review board and clinical research ethics committee.

Definitions were the following: (1) Diagnosis of absent NB in the first trimester is considered when NB is not visualized on a mid-sagittal view of the profile. (2) Diagnosis of absent NB in the second trimester was considered when NB is not visualized on any appropriate view (including sagittal, transverse and coronal sections). (3) Diagnosis of hypoplastic NB in the second trimester was defined as one or both sides of the nasal bone length below the 2.5th percentile of Chinese population [16], including those with unilateral absence of nasal bone.

Following prenatal detection of an absent NB in the first trimester, detailed anomaly scans were scheduled in the second trimester to confirm persistence of it and assess for associated anomalies. Foetus was considered isolated if no other associated anomalies including soft markers (such as increased nuchal translucency (NT)) or structural abnormality were noted.

**Figure 1.**
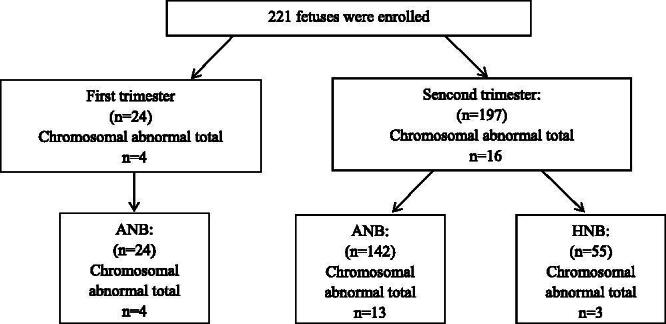
Prenatal diagnosis for foetuses with prenatally diagnosed with isolated absent or hypoplastic nasal bone.

Following prenatal detection of an absent or hypoplastic foetal NB in the second trimester, a systematic sonographic assessment for associated anomalies was performed. Foetus was considered isolated if no other associated anomalies including soft markers (such as single umbilical artery) or structural abnormality were noted. All sonographic findings were confirmed by experienced sonologists dedicated to obstetric sonography. Therefore, the classifications in this study are based specifically upon the phenotype at initial presentation before invasive prenatal diagnosis (Figure 1).

Prenatal genetic testing was recommended for the parents, and the potential benefit and risk of invasive prenatal diagnosis and CMA were explained. Written informed consent was obtained before invasive test.

Microarray analyses were performed using a high-resolution genotyping single nucleotide polymorphism microarray, Affymetrix CytoScan 750 K Array (Affymetrix, Santa Clara, CA, USA). CNVs were identified based on associated records of the human reference genome 37(NCBI37hg19) of the National Centre for Biotechnology Information. Data were analyzed in accordance with American College of Medical Genetics guidelines.

### Statistical analysis

Quantitative variables are expressed as the mean ± standard deviation, and categorical variables are expressed as the frequency and percentage. Fisher’s Exact test or *χ*^2^ were used to test the differences between CMA yield in relation to different parameters and compared to the background risk. *p* < .05 was considered statistically significant.

## Results

There were 1267 foetuses with prenatally diagnosed with isolated absent or hypoplastic NB during the study period. Totally, 221 cases chose invasive prenatal diagnosis by CMA, including 166 cases with isolated absent NB and 55 cases with isolated hypoplastic NB. Twenty-four foetuses (10.9%, 24/221) had an ultrasonic diagnosis in the first trimester and 197 (89.1%, 197/221) had an ultrasonic diagnosis in the second trimester. The mean maternal age was 24.0 ± 4.0 years. Mean gestational age at diagnosis was 29.4 ± 5.3 weeks. Overall, 3.6% (8/221) of prenatal samples were performed following chorionic villus sampling, 67.4% (149/221) from amniocentesis and 29.0% (64/221) from cordocentesis sampling.

The overall diagnostic yield of CMA testing for foetuses with isolated absent or hypoplastic NB was 9.0% (20/221). Aneuploidies were detected in 13 (5.9%, 13/221) foetuses, including 10 Down syndrome (4.5%, 10/221), 2 Klinefelter's syndrome (0.9%, 2/221) and 1 trisomy 18 (0.5%, 1/221) (Table 1). Pathogenic CNVs were detected in seven foetuses (3.2%, 7/221). In addition, VOUS were detected in 4 foetuses (1.8%, 4/221). The details of the identified pathogenic CNVs and VOUS are presented in Tables 2 and 3.

**Table 1.** Aneuploidies in foetuses with isolated absent or hypoplastic nasal bone.

NO	MA	GA	Ultrasonic diagnosis	Trimester of ultrasonic diagnosis	CMA results	Pregnant outcomes
1	39	13	ANB	First trimester	Trisomy 21	TOP
2	39	17	ANB	First trimester	Trisomy 21	TOP
3	22	22	ANB	First trimester	Trisomy 21	TOP
4	36	25	ANB	Second trimester	Trisomy 21	TOP
5	41	27	ANB	Second trimester	Trisomy 21	TOP
6	45	19	ANB	Second trimester	Trisomy 21	TOP
7	28	28	ANB	Second trimester	Trisomy 21	TOP
8	23	22	ANB	Second trimester	Trisomy 21	TOP
9	38	17	ANB	Second trimester	Trisomy 21	TOP
10	38	17	ANB	Second trimester	Trisomy 21	TOP
11	28	19	ANB	First trimester	Trisomy 18	TOP
12	26	25	ANB	Second trimester	47, XXY	TOP
13	30	17	ANB	Second trimester	47, XXY	Caesarean section at 40w, male, 3.56 kg, Normal newborn examination

*Abbreviations:* MA: mean maternal age at which the invasive prenatal diagnosis was performed; GA: gestational age at which the invasive prenatal diagnosis was performed. ANB: absent nasal bone; TOP: termination of pregnancy.

**Table 2.** Pathogenic CNVs in foetuses with isolated absent or hypoplastic nasal bone.

NO	MA	GA	Ultrasonic diagnosis	Trimester of ultrasonic diagnosis	CMA results	Size of CNVs	Outcomes
1	34	28	ANB	Second trimester	Xp22.33 or Yp11.32(522,089–1,234,634 or 472,089–1,184,634)×1	713 kb	Inherited from father, eutocia section at 40^+1^w, male, 3.4 kg. Normal newborn examination
2	30	26	ANB	Second trimester	14q22.1q22.3(53,708,880–56,732,343)×1	3.0 Mb	de novo, TOP
3	26	24	ANB	Second trimester	6p21.1p12.3(45,406,978–49,520,083)×1	4.1 Mb	TOP
4	18	28	ANB	Second trimester	1q21.1q21.2(146,106,724–147,391,923)×3	1.3 Mb	Eutocia section at 38^+5^w, female, 3.0 kg. Normal newborn examination
5	31	30	HNB	Second trimester	Xp22.31(6,449,752–8,143,319)×1	1.7 Mb	Ceaserian section at 40^+2^w, female, 3.45 kg. Normal newborn examination
6	31	25	HNB	Second trimester	1p36.33p36.31(849,466–5,708,006)×1	4.9 Mb	TOP
7	29	24	HNB	Second trimester	16p11.2(29,428,531–30,190,029)×1	761 kb	Eutocia section at 39^+5^w, female, 2.88 kg. Normal newborn examination

*Abbreviations:* MA: mean maternal age at which the invasive prenatal diagnosis was performed; GA: gestational age at which the invasive prenatal diagnosis was performed; ANB: absent nasal bone; HNB: hypoplastic nasal bone; TOP: termination of pregnancy.

**Table 3.** VOUS in foetuses with isolated absent or hypoplastic nasal bone.

NO	MA	GA	Ultrasonic diagnosis	trimester of ultrasonic diagnosis	CMA	Size of CNVs	Outcomes
1	27	24	ANB	Second trimester	12q21.31(83,844,899–86,010,755)×3,	2.17 Mb	TOP
					Xp22.31p22.2(9,219,703–10,190,141)×2	970 kb	
2	26	24	ANB	Second trimester	5q23.1(118,643,271–119,998,736)×3	1.3 Mb	Inherited from father, eutocia section at 39 + 2w, female, 3.0 kg
3	26	25	HNB	Second trimester	8q24.11(118,170,260–118,875,304)×3	705 kb	Inherited from father and mother, eutocia section at 39 + 5w, male, 3.35 kg
					Xp11.22(52,341,517–52,911,475)×0	570 kb	
4	26	24	HNB	Second trimester	16p13.11(15,481,747–16,309,046)×3	827 kb	Eutocia section at 37 + 3w, male, 2.7 kg

*Abbreviations:* MA: mean maternal age at the invasive prenatal diagnosis was performed; GA: gestational age at the invasive prenatal diagnosis was performed; ANB: absent nasal bone; HNB: hypoplastic nasal bone; TOP: termination of pregnancy.

Seven pathogenic CNVs (Table 2) sized from 713 kb to 4.9 Mb. The pathogenic CNVs were included Xp22.33 or Yp11.32 microdeletion, 14q22.1q22.3 microdeletion, 6p21.1p12.3 microdeletion, 1q21.1 microduplication, Xp22.31 microdeletion, 1p36 microdeletion and 16p11.2 microdeletion (Table 2).

In 166 foetuses with isolated absent NB, there were 13 cases of aneuploidies and 4 cases of pathogenic CNVs, and the chromosome abnormality rate was 10.2% (17/166). There were three cases of pathogenic CNVs in isolated hypoplastic NB. The foetuses with isolated absent NB had a higher detection rate of chromosome abnormality than the isolated hypoplastic NB group, but the difference was not significant in the statistical analysis (10.2% vs. 5.5% %, *χ*^2 ^=0.642, *p* = .423).

Four foetuses (4/24, 16.7%) with chromosomal abnormalities were identified in the first trimester, all were aneuploidies. Sixteen foetuses with chromosomal abnormalities (16/197, 8.1%) were detected in the second trimester, including nine aneuploidies and seven pathogenic CNVs. No significant difference was observed in the detection rate between the first trimester and the second trimester (16.7% vs. 8.1%, *χ*^2 ^=1.002, *p* = .317).

## Discussion

Previous studies have demonstrated that the absence or hypoplasia of NB may be one of the strongest ultrasound markers for Down syndrome and other chromosomal abnormalities in both the first and second trimesters. However, studies of foetuses with isolated absent or hypoplastic NB by CMA were limited. We specifically focussed on CMA as first-tier testing in foetuses with isolated absent or hypoplastic NB and attempted to elaborate the relationship between pathogenic CNVs and isolated absent or hypoplastic NB. We identified chromosomal abnormalities in 9.0% foetuses, including 13 aneuploidies and 7 pathogenic CNVs. Down syndrome was the most common chromosomal abnormalities in our study and detected in 10 (4.5%) foetuses. This result further supports the idea that absent or hypoplastic foetal NB is a strong marker for Down syndrome.

Despite an increased diagnostic yield, the use of CMA in foetuses with isolated absent or hypoplastic NB is still controversial. Lostchuck et al. reported 80 cases for isolated hypoplastic NB. There were no cases of pathogenic CNVs in the 47/80 cases that were analyzed by CMA [14]. In contrast, Gu et al. reported their tertiary-centre experience with isolated hypoplastic NB and found a non-trisomy-21 abnormality in 2 of the 39 foetuses, resulting in a frequency of pathogenic CNVs in isolated hypoplastic NB of 5.1% [15]. Du et al. reported 42 cases with isolated absence NB during the second trimester and found 1 had a microdeletion [17]. Wu et al. studied 111 cases with abnormal foetal NB (59 cases with absence NB and 52 cases with hypoplastic NB) and found 4 foetuses with pathogenic CNVs (3.6%, 4/111)[18]. Huang et al. reported 32 cases with isolated hypoplastic NB, and found three foetuses with pathogenic CNVs and four with VOUS [12]. To our best knowledge, this study is the first report describing the rate of chromosome abnormality in foetuses with isolated absent or hypoplastic NB. Aside from aneuploidy, CMA showed seven pathogenic CNVs, for a detection rate of 3.2%. It has been reported by Callaway et al. that pathogenic/likely pathogenic CNVs were detected in approximately 1.0% (94/9272) of the foetuses with normal ultrasound examination results and normal karyotyping [19]. The diagnostic yield of CMA for pathogenic CNVs in foetuses with isolated absent or hypoplastic NB in our study was 3.2%, which is much higher than the control population (*χ*^2 ^=10.673, *p* = .001, Chi-square test). Thus, CMA can increase the diagnostic yield of chromosomal abnormalities for foetuses with isolated absent or hypoplastic NB.

Cell-free DNA or so-called non-invasive prenatal testing (NIPT) is now widely used in clinical practice as a prenatal screening method for common aneuploidies. It also has the potential to detect foetal CNVs, but with false-positive and false-negative results. Recent studies have showed that the accuracy of NIPT for CNVs is still unsatisfactory and needs to be improved [20,21]. CMA is still the most effective method for CNVs detection. On the other hand, conventional karyotyping can identify the majority of foetal chromosomal abnormalities, at a resolution of greater than 10 Mb. In this study, the deletion or duplication sizes of the seven cases were ranged from 713 kb to 4.9 Mb, and all of them may not be diagnosed by the G-banding karyotyping. Finally, from a patient's point of view, they want to exclude as many abnormalities as possible to ensure that the foetus is healthy. Therefore, for prenatal genetic counselling, counsellors should discuss the possibility of pathogenic CNVs to parents when foetuses with isolated absent or hypoplastic NB. CMA should be recommended when isolated absent or hypoplastic NB is suspected antenatally.

Fantasia et al. analyzed the association of first trimester absent NB and genetic abnormalities at G-banding karyotype and CMA according to the NT thickness. The results showed pathogenic CNVs were found only in the group with NT > 99th centile [22]. In this study, we also found no pathogenic CNVs in the first trimester isolated absent NB. These results demonstrated no increase in the risk for pathogenic CNVs in cases with isolated absent NB in the first trimester. However, due to the small sample size, prospective well-adjusted studies are needed to guide the optimal management of these foetuses. Zhang et al. compared 35 cases with isolated absent NB and 20 cases with isolated hypoplastic NB, and found the chromosomal abnormalities rate was increased in the foetuses with isolated hypoplastic NB [15]. However, in our study, the foetuses with isolated absent NB had a higher detection rate of chromosomal abnormalities than the isolated hypoplastic NB, although the difference was not significant in the statistical analysis. The possible reasons for the discrepancy between two studies were the considerably little sample size in the previous study (55 vs. 229 foetuses) and the difference of inclusion criteria (just second trimester vs. first and second trimester).

Seven cases were reported as pathogenic CNVs and the fragment sizes ranged from 713 kb to 4.9 Mb. In case 4, a duplication of 1.3 Mb at distal 1q21.1 region was found. The phenotype of duplication of 1q21.1 region is variable, ranging from macrocephaly, autism spectrum disorder, congenital anomalies, to a normal phenotype. In this study, after comprehensive genetic counselling, the couples ultimately chose to continue the pregnancy. Ji et al. reported three foetuses with 1q21.1 duplication and found two with NB loss, indicating absent foetal NB may be related to 1q21.1 duplication [23]. Zhang et al. also reported 55 foetuses with isolated absent or hypoplastic NB and found one foetus with 1q21.1 duplication [15]. Combined with the three cases of the previous studies and our research, these results identify a further expansion of the prenatal presentation of 1q21.1 duplication. Accordingly, there may be a connection between this duplication and absent or hypoplastic NB, although this remains to be proven by an enlarged study.

Several additional limitations of the current study should be acknowledged. The clinical data are based on a single-centre, and were collected retrospectively. Follow-up data of long-term after childbirth were unavailable.

## Conclusion

In summary, the results of the present study demonstrate that CMA can increase the diagnostic yield of chromosome abnormality for foetuses with isolated absent or hypoplastic NB. Counsellors should discuss the possibility of pathogenic CNVs to parents when foetuses with isolated absent or hypoplastic NB. CMA should be recommended when isolated absent or hypoplastic NB is suspected antenatally.

## Data Availability

All data included in this study are available upon request by contact with the corresponding author.

## References

[1] Moreno-Cid M, Rubio-Lorente A, Rodríguez MJ, et al. Systematic review and meta-analysis of performance of second-trimester nasal bone assessment in detection of fetuses with down syndrome. Ultrasound Obstet Gynecol. 2014;43(3):247–253. 24151178 10.1002/uog.13228

[2] Agathokleous M, Chaveeva P, Poon LC, et al. Meta-analysis of second-trimester markers for trisomy 21. Ultrasound Obstet Gynecol. 2013;41(3):247–261. 23208748 10.1002/uog.12364

[3] Cicero S, Rembouskos G, Vandecruys H, et al. Likelihood ratio for trisomy 21 in fetuses with absent nasal bone at the 11–14 week scan. Ultrasound Obstet Gynecol. 2004;23(3):218–223. 15027007 10.1002/uog.992

[4] Dukhovny S, Wilkins-Haug L, Shipp T, et al. Absent fetal nasal bone: what does it mean for the euploid fetus?. J Ultrasound Med. 2013;32(12):2131–2134. 24277895 10.7863/ultra.32.12.2131

[5] Sherer DM, Eugene P, Dalloul M, et al. Second-trimester diagnosis of cri du chat (5p-) syndrome following sonographic depiction of an absent fetal nasal bone. J Ultrasound Med. 2006;25(3):387–388. 16495500 10.7863/jum.2006.25.3.387

[6] Xing Y, Holder J, Liu Y, et al. Prenatal diagnosis of Wolf-Hirschhorn syndrome: from ultrasound findings, diagnostic technology to genetic counseling. Arch Gynecol Obstet. 2018;298(2):289–295. 29808250 10.1007/s00404-018-4798-1

[7] Wapner RJ, Martin CL, Levy B, et al. Chromosomal microarray versus karyotyping for prenatal diagnosis. N Engl J Med. 2012;367(23):2175–2184. 23215555 10.1056/NEJMoa1203382 PMC3549418

[8] Hillman SC, McMullan DJ, Hall G, et al. Use of prenatal chromosomal microarray: prospective cohort study and systematic review and meta-analysis. Ultrasound Obstet Gynecol. 2013;41(6):610–620. 23512800 10.1002/uog.12464

[9] Miller DT, Adam MP, Aradhya S, et al. Consensus statement: chromosomal microarray is a first-tier clinical diagnostic test for individuals with developmental disabilities or congenital anomalies. Am J Hum Genet. 2010;86(5):749–764. 20466091 10.1016/j.ajhg.2010.04.006 PMC2869000

[10] Ting YH, Lao TT, Lau T, et al. Isolated absent or hypoplastic nasal bone in the second trimester fetus: is amniocentesis necessary? J Matern Fetal Neonatal Med. 2011;24(4):555–558. 21375370 10.3109/14767058.2010.487140

[11] Du Y, Ren Y, Yan Y, et al. Absent fetal nasal bone in the second trimester and risk of abnormal karyotype in a prescreened population of Chinese women. Acta Obstet Gynecol Scand. 2018;97(2):180–186. 29164604 10.1111/aogs.13263 PMC5814939

[12] Huang H, Cai M, Ma W, et al. Chromosomal microarray analysis for the prenatal diagnosis in fetuses with nasal bone hypoplasia: a retrospective cohort study. Risk Manag Healthc Policy. 2021;14:1533–1540. 33889037 10.2147/RMHP.S286038 PMC8054820

[13] Hou l, Wang x, Jiang h, et al. Application of chromosomal analysis for 29 cases of fetuses with nasal bone absence or hypoplasia. Zhonghua Yi Xue Za Zhi. 2018;98(43):3532–3535. 30481905 10.3760/cma.j.issn.0376-2491.2018.43.014

[14] Lostchuck E, Hui L. Should second trimester hypoplastic nasal bone be sole indication for diagnostic testing with chromosomal microarray analysis? Ultrasound Obstet Gynecol. 2019;53(6):848–850. 30302840 10.1002/uog.20141

[15] Zhang F, Long W, Zhou Q, et al. Is prenatal diagnosis necessary for fetal isolated nasal bone absence or hypoplasia? Int J Gen Med. 2021;14:4435–4441. 34408481 10.2147/IJGM.S322359 PMC8364966

[16] Hung JH, Fu CY, Chen CY, et al. Fetal nasal bone length and down syndrome during the second trimester in a Chinese population. J Obstet Gynaecol Res. 2008;34(4):518–523. 18946935 10.1111/j.1447-0756.2008.00747.x

[17] Gu YZ, Nisbet DL, Reidy KL, et al. Hypoplastic nasal bone: a potential marker for facial dysmorphism associated with pathogenic copy number variant on microarray. Prenat Diagn. 2019;39(2):116–123. 30578730 10.1002/pd.5410

[18] Wu LJ, Cao L, Hu P, et al. Research on the ultrasonographic diagnosis of fetal nasal bone dysplasia and choromsome microarray analysis results. Chin J Ultrasound Med. 2021;5(37):567-570.

[19] Callaway JL, Shaffer LG, Chitty LS, et al. The clinical utility of microarray technologies applied to prenatal cytogenetics in the presence of a normal conventional karyotype: a review of the literature. Prenat Diagn. 2013;33(12):1119–1123. 23983223 10.1002/pd.4209 PMC4285999

[20] Duan HL, Li J, Wang WJ, et al. Cell-free DNA test for pathogenic copy number variations: a retrospective study. Taiwan J Obstet Gynecol. 2021;60(6):1066–1071. 34794739 10.1016/j.tjog.2021.09.018

[21] Pei Y, Hu L, Liu J, et al. Efficiency of noninvasive prenatal testing for the detection of fetal microdeletions and microduplications in autosomal chromosomes. Mol Genet Genomic Med. 2020;8(8):e1339. 32543126 10.1002/mgg3.1339 PMC7434727

[22] Stampalija T, Sirchia F, Della Pietà I, et al. First-trimester absent nasal bone: is it a predictive factor for pathogenic CNVs in the low-risk population? Prenat Diagn. 2020;40(12):1563–1568. 32799336 10.1002/pd.5812

[23] Ji X, Pan Q, Wang Y, et al. Prenatal diagnosis of recurrent distal 1q21.1 duplication in three fetuses with ultrasound anomalies. Front Genet. 2018;9(9):275. 30177949 10.3389/fgene.2018.00275 PMC6109635

